# Metabolomic Profiling of Bile Acids in Clinical and Experimental Samples of Alzheimer’s Disease

**DOI:** 10.3390/metabo7020028

**Published:** 2017-06-17

**Authors:** Xiaobei Pan, Christopher T. Elliott, Bernadette McGuinness, Peter Passmore, Patrick G. Kehoe, Christian Hölscher, Paula L. McClean, Stewart F. Graham, Brian D. Green

**Affiliations:** 1 Advanced Asset Technology Centre, Institute for Global Food Security, Queen’s University Belfast, Belfast BT9 5AG, UK; x.pan@qub.ac.uk (X.P.); Chris.Elliott@qub.ac.uk (C.T.E.); 2 Centre for Public Health, School of Medicine, Dentistry and Biomedical Sciences, Queen’s University Belfast, Belfast BT12 6BA, UK; B.McGuinness@qub.ac.uk (B.M.); P.Passmore@qub.ac.uk (P.P.); 3 Dementia Research Group, Institute of Clinical Neurosciences, School of Clinical Sciences, University of Bristol, Bristol BS10 5NB, UK; patrick.kehoe@bristol.ac.uk; 4 Division of Biomedical and Life Sciences, Lancaster University, Lancaster LA1 4YG, UK; c.holscher@lancaster.ac.uk; 5 Biomedical Sciences Research Institute, Ulster University, Clinical Translational Research and Innovation Centre C-TRIC, Derry/Londonderry BT47 6SB, UK; pl.mcclean@ulster.ac.uk; 6 Beaumont Research Institute, Beaumont Health, Royal Oak, MI 48073, USA; Stewart.Graham@beaumont.org

**Keywords:** bile acids, metabolomics, Alzheimer’s disease, metabolites

## Abstract

Certain endogenous bile acids have been proposed as potential therapies for ameliorating Alzheimer’s disease (AD) but their role, if any, in the pathophysiology of this disease is not currently known. Given recent evidence of bile acids having protective and anti-inflammatory effects on the brain, it is important to establish how AD affects levels of endogenous bile acids. Using LC-MS/MS, this study profiled 22 bile acids in brain extracts and blood plasma from AD patients (*n* = 10) and age-matched control subjects (*n* = 10). In addition, we also profiled brain/plasma samples from APP/PS1 and WT mice (aged 6 and 12 months). In human plasma, we detected significantly lower cholic acid (CA, *p* = 0.03) in AD patients than age-matched control subjects. In APP/PS1 mouse plasma we detected higher CA (*p* = 0.05, 6 months) and lower hyodeoxycholic acid (*p* = 0.04, 12 months) than WT. In human brain with AD pathology (Braak stages V-VI) taurocholic acid (TCA) were significantly lower (*p* = 0.01) than age-matched control subjects. In APP/PS1 mice we detected higher brain lithocholic acid (*p* = 0.05) and lower tauromuricholic acid (TMCA; *p* = 0.05, 6 months). TMCA was also decreased (*p* = 0.002) in 12-month-old APP/PS1 mice along with 5 other acids: CA (*p* = 0.02), β-muricholic acid (*p* = 0.02), Ω-muricholic acid (*p* = 0.05), TCA (*p* = 0.04), and tauroursodeoxycholic acid (*p* = 0.02). The levels of bile acids are clearly disturbed during the development of AD pathology and, since some bile acids are being proposed as potential AD therapeutics, we demonstrate a method that can be used to support work to advance bile acid therapeutics.

## 1. Introduction

Bile acids play complex roles in cell signalling and immunomodulation, and bile acid receptors are considered therapeutic targets for various metabolic diseases [1,2]. Primary bile acids are synthesized in liver cells through the oxidation of cholesterol. They play an important role in lipid digestion. The oxidized cholesterol metabolites, oxysterols, activate nuclear receptors (LXRs) which are implicated in neurodegenerative disease [3,4]. Greenberg et al. observed a trend of increased glycocholic acid (GCA), glycodeoxycholic acid (GDCA), and glycochenodeoxycholic acid (GCDCA) in mild cognitive impairment (MCI) and AD, although none of these reached statistical significance [5]. Just recently, it has been reported that the blood plasma levels of deoxycholic acid (DCA), lithocholic acid (LCA), and GDCA acids are significantly elevated in patients with amnestic MCI and AD [6]. Similarly, significant increases in the levels of the secondary bile acid glycoursodeoxycholic acid (GUDCA) have been described in the plasma of AD patients [7].

Considerably fewer studies have examined the levels of bile acids in the brain, but the occurrence of these molecules has been reported [8,9,10]. For example, cholic acid (CA), chenodeoxycholic acid (CDCA), and DCA have been detected in rat brain [8]. The levels of CDCA in the brain were approximately 30 times greater than those in serum [8]. Some of the underlying biochemistry has been established. For example, the conversion of 3β-hydroxychol-5-en-24-oic acid to CDCA via 3β,7α-dihydroxychol-5-en-24-oic acid and 7α-hydroxy-3-oxochol-4-en-24-oic acid has been demonstrated in the rat brain [9].

There also appears to be some scope to apply bile acids as potential AD therapeutics [11,12]. One line of inquiry is based upon the strong modulatory effect of some bile acids on apoptosis and the observation that tauroursodeoxcholic acid (TUDCA) can inhibit neuronal apoptosis in a number of experimental models of neurodegenerative disease. TUDCA appears to be neuroprotective in AD, Huntington’s disease (HD), and Parkinson’s disease (PD) [11]. In models of AD pathology TUDCA reduces p53-mediated apoptosis in AD mutant neuroblastoma cells [11]. Systemic administration of TUDCA significantly reduces striatal neuropathology in the R6/2 transgenic HD mouse [12]. TUDCA improves the survival and function of nigral transplants in a rat model of Parkinson’s disease [13]. Furthermore, in an animal model of acute neuro-inflammation TUDCA has a ‘triple anti-inflammatory’ effect on the glial cells [14]. It reportedly reduces glial cell activation, reduces microglial cell migratory capacity, and lowers expression of chemoattractants and vascular adhesion proteins [14].

The rapid development of metabolomics as a discipline now makes it much more feasible to profile the wide range of bile acid species which occur physiologically. A range of metabolomic and lipidomic investigations of human AD and AD-like pathology have been undertaken. For example, studies have undertaken non-targeted profiling of polar metabolites [15,16,17,18] and non-polar (lipid) metabolites [19], and there has also been targeted profiling of fatty acids [20,21], phospholipids [7,22], and steroids [23]. However, no study as far as we are aware has focused exclusively on the profiling of bile acids. The fact that some non-targeted metabolomic studies have reported disturbed levels of some bile acids in AD is encouraging, but it should be pointed out that, to date, there has not been any focused examination of how AD pathology affects brain bile acids. Measuring changes in brain levels would seem to be an appropriate issue to address, given the observed protective effects of these molecules (i.e., TUDCA) on the brain.

This study profiled a range of conjugated and unconjugated C24 bile acids across a range of AD samples. We sampled blood plasma from AD patients and cognitively normal aged-matched control subjects, as well as neocortical tissue from pathologically confirmed cases of AD and age-matched controls. We also sampled blood plasma and brain samples from APP/PS1 mice and WT littermates at both 6 months and 12 months of age. Our hypothesis was that the bile acid system is affected by the development of AD pathology and the aim was to determine specific bile acid changes. 

## 2. Results

### 2.1. Bile Acid Levels in Human Plasma

Of the 22 bile acids screened, 15 were quantifiable in the human plasma samples analysed. Only CA was significantly affected in cases of AD (Table 1). In AD patients CA was significantly lower (947 ± 483 nM vs. 156 ± 74 nM; *p* = 0.03). 

### 2.2. Bile Acid Levels in Mouse Plasma

Of the 22 bile acids screened, 14 were quantifiable in mouse plasma. Only CA and HDCA were significantly different between APP/PS1 and WT mice (Table 2). In 6-month-old APP/PS1 mice we detected higher CA (1811 ± 368 nM vs. 10510 ± 4498 nM; *p* = 0.05), however this was not apparent at 12 months. Similarly, we detected a significant decrease in HDCA in 12-month-old APP/PS1 mice which was not evident at 6 months of age (1009 ± 226 nM vs. 364 ± 192 nM; *p* = 0.04).

### 2.3. Bile Acid Levels in Human Brain

Of the 22 bile acids screened, only 11 were quantifiable in the human brain specimens analysed. Only TCA was significantly different in AD cases (Table 3). In samples with AD pathology, TCA was significantly lower (0.01 ± 0.006 nmol/g vs. 0.06 ± 0.02 nmol/g; *P* = 0.01) than in age-matched control subjects.

### 2.4. Bile Acid Levels in Mouse Brain

Of the 22 bile acids screened, only 8 were quantifiable in mouse brain tissue. At 6 months of age, only 2 bile acids were significantly different, however, at 12 months a total of 6 bile acids differed between APP/PS1 and WT mice (Table 4). In 6-month-old APP/PS1 mice we detected higher brain LCA (0.04 ± 0.01 nmol/g vs. 0.01 ± 0.01 nmol/g; *p* = 0.05) and lower TMCA (0.08 ± 0.03 nmol/g vs. 0.14 ± 0.02 nmol/g; *p* = 0.05). TMCA was also decreased in 12 month old APP/PS1 mice (0.04 ± 0.01 nmol/g vrs; 0.25 ± 0.07 nmol/g; *p* = 0.002) along with 5 others: CA (0.08 ± 0.02 nmol/g vs. 0.28 ± 0.08 nmol/g; *p* = 0.02), β-MCA (0.08 ± 0.04 nmol/g vs. 0.24 ± 0.06 nmol/g; *p* = 0.02), Ω-MCA (0.03 ± 0.01 nmol/g vs. 0.08 ± 0.02 nmol/g; *p* = 0.05), TCA (0.04 ± 0.02 nmol/g vs. 0.16 ± 0.07 nmol/g; *p* = 0.04), and TUDCA (0.01 ± 0.0.001 nmol/g vs 0.03 ± 0.01 nmol/g; *p* = 0.02).

## 3. Discussion

This pilot study employed a targeted LC-MS/MS metabolomics methodology to profile 22 bile acids in blood plasma from AD patients and human brain specimens of pathologically confirmed cases of AD. We also profiled blood plasma and brain tissue from APP/PS1 mice with ‘early’ (6 months) and “late” (12 months) AD-like pathology. This is the first metabolomic study to focus exclusively on bile acid changes in AD, including the study of these in brain, either in human AD or in an experimental model of AD.

A non-targeted investigation by Olazaran et al. found plasma bile acid disturbances in persons with amnestic MCI and AD, including changes in DCA, LCA, and GDCA [6]. In both amnestic MCI and AD, these 3 bile acids were increased [6]. Significant increases in the levels of the secondary bile acid GUDCA were reported in the plasma of AD patients [7]. However, in the present study none of these bile acids were elevated in human plasma from AD patients. Only one bile acid, CA, was significantly lower in AD patients than in age-matched control subjects. CA was also disturbed in plasma and brain from APP/PS1 mice. Here we report brain CA to be more than 3-fold lower in the transgenic mouse than in the WT, but, in contrast, plasma CA was significantly higher. 

The APPswe/PS1deltaE9 (APP/PS1) double transgenic mouse has been extensively employed in AD research for a number of years. The model develops detectable Aβ plaques in the brain by 5–6 months of age, and the mice display progressive age-related impairments in memory that appear as early as 7 months [24,25]. Behavioural testing shows that the mice have deficits in their spatial navigation and in reference learning [26]. When we measured bile acid levels in the brain extracts from APP/PS1 and WT mice that were 6 months old we only found two disturbances: LCA, which more than doubled, and TMCA, that approximately halved. In 12-month-old APP/PS1 mice the disturbances were more wide-ranging, with a total of 6 bile acids being affected. In all cases the levels in APP/PS1 mice were lower. TMCA was 6-fold lower, TCA was 4-fold lower, CA, β-MCA, and Ω-MCA and TUDCA were 3-fold lower. 

As expected, we saw differing bile acid profiles between mouse and human samples [26]. For example, HDCA, α-MCA, and β-MCA were all absent in human plasma but present in mouse plasma, and there were a host of bile acids that were present in human plasma but absent in mouse. There were also substantial differences in the concentration ranges of common bile acids. For example, plasma CA and DCA were substantially higher in mouse plasma than in human plasma. Fewer bile acids were detectable in brain tissue and those that were detectable were generally observed to be much lower. As previously stated, there has been very little investigation of the brain levels of bile acids, and the findings of the present study conflict with a previous report [8]. In the earlier study, it was reported that CDCA levels in rat brain were approximately 30 times greater than those in serum [8]. In fact, the opposite was true in the present study, with CDCA being undetectable in mouse brain. It also was previously reported that CDCA levels were around 30 times higher than CA and DCA [8], which was also not the case here. This could relate to rat/mouse species differences in bile acid metabolism [26] and demonstrates the need to elucidate the neurological levels and functions of these molecules. However, we do note one consistency with the studies of Mano et al. on the rat brain [8]. They were unable to detect any glycine-conjugated bile acids in rat brain and we found these to be similarly below the limit of detection in the mouse brain. There are contrasting differences between human and mice specimens. For example, CA was significantly lower in AD patients’ plasma, but higher in 6-month-old APP/PS1 mouse plasma. These differences could be explained in a number of ways. Firstly, APP/PS1 mice exhibit a particular aspect of AD pathology and not the many complex features of the human disease. The transgenic model is free from the influences and variability caused by differing medication and dietary habits of humans and this could be a factor. Finally, it is worth remembering that there are inherent metabolic differences between mice and humans which mean that they do not possess entirely the same complement of bile acids, and even though both species produce CA and TCA, the other metabolic pathways could impact on them. TCA was significantly lower in both the human AD brain and the 12-month-old APP/PS1 mouse brain.

A common feature associated with the measurement of bile acids is the inherently large variances observed, and this may limit their use as biomarkers, at least in AD [5]. Here, the variances were particularly large even in the brain that is a largely self-contained and tightly regulated organ. We observed coefficients of variation as high as 75%. There are a number of extraneous variables that modulate bile acid levels. Besides the potential variations in their biosynthesis in the liver (which is impacted by dietary factors and cholesterol metabolism) there is also the much more unpredictable influence of the gut microbiota that transforms bile acids. Conditions such as dysbiosis (pathological imbalance in a microbial community) can significantly impair the metabolism of bile acids and may result in an inability to maintain glucose homeostasis as well as normal cholesterol breakdown and excretion [27]. Similarly, antibiotic treatment causes levels of secondary bile acids to decrease, whereas those of primary bile acids increase as a consequence of the modified metabolic activity of the altered gut microbiome [28]. Bile metabolism is therefore impacted by the gut microbiome and this is difficult to account for in the present studies. This study was a pilot which has allowed us to rapidly assess to what extent bile acids are impacted by the development of AD pathology. However, as with many pilot investigations, there are limitations. Firstly, it was not possible to account for differences in the medication or diet of the human subjects and this remains a potential confounding factor in this study. Secondly, ageing also has a profound effect on cholesterol metabolism which could influence the biosynthesis of bile acids. Thirdly, the relatively small sample size and student’s T-test that were used in this pilot study may result in false-positives or in over-estimating the magnitude of changes [29]. It should be acknowledged that applying more stringent statistical approaches (incorporating false-discovery rates or Bonferroni corrections) to these datasets inevitably lead to a lack of significant differences. Therefore, key recommendations for future studies investigating correlations between AD and bile acids should be that they try to mitigate the large variances by greatly increasing sample size, applying more stringent statistical approaches which reduce type I errors, and controlling or accounting for the variations in gut microflora, medication, ageing, gender, and diet. 

For future research, there also could be merit in examining the C27 bile acid-intermediates which are poorly characterised compared with the C24 bile acids measured here. In plasma, C27 bile acids can exceed the levels of C24 bile acids and, promisingly, there appears to be smaller variations between individuals [30]. C27 bile acid-intermediates have been measured in the human brain [10]. Interestingly, the concentrations of the C27 bile acid 7α-hydroxy-3-oxocholest-4-en-26-oic acid change substantially in CSF following surgery for aneurysmal subarachnoid hemorrhage [31], and the levels of this bile acid are higher in chronic subdural hematoma [32]. 7α-hydroxy-3-oxocholest-4-en-26-oic acid has been shown to be the major cholesterol metabolite found in human CSF and an intermediate in the biosynthesis of other bile acids [4]. C27 bile acid-intermediates are more hydrophobic and potentially more cytotoxic than C24 products and the examination of these less well-characterised metabolites in AD could be worthwhile [10]. 

## 4. Materials and Methods

### 4.1. Human Plasma and Post-Mortem Brain Tissue

Plasma samples were obtained from AD patients (*n* = 10) and healthy age-matched controls (*n* = 10) recruited from the Belfast City Hospital memory clinic. The diagnosis of AD was made using the NINCDS-ADRDA criteria [33]. Plasma was collected in EDTA tubes from individuals using standard venepuncture procedures. Samples were immediately centrifuged and stored in aliquots at −80 °C until the date of analysis. Subjects were as closely age-matched as possible. Demographic characteristics are summarised in Table 5. Participant demographics and clinical characteristics are detailed in Supplementary Table S1.

Brain tissue samples (neocortex, Brodmann area 7) were obtained from postmortem confirmed AD cases (*n* = 10) and elderly non-demented age-matched control subjects (*n* = 10). Tissue cases were geographically spread across the UK (Bristol, Newcastle, and London) and were obtained through the Brains for Dementia Research (BDR) initiative, a brain bank network funded by ARUK and co-ordinated from King’s College London. Consent and ethical approval for the use of tissue was obtained by individual brain banks, all of which are licensed by the Human Tissue Authority. Demographic characteristics are summarised in Table 5. Participant characteristics such as gender, age, PM delay, and Braak stage are detailed in Supplementary Table S2.

### 4.2. Mouse Plasma and Brain Tissue

Founder APPswe/PS1DE9 (APP/PS1) male mice were initially obtained from the Jackson lab (USA) and bred at the Ulster University. Heterozygous males were bred with wild-type (WT) C57/Bl6 females bought locally (Harlan, UK). APP/PS1and WT mice were housed under identical conditions and fed the same rodent maintenance diet (14% fat, 32% protein, and 54% carbohydrate; total energy of 3.0 kcal/g; Harlan). All experiments were licensed by the UK Home Office (project licence number PPL2734) in accordance with the Animal (scientific procedures) Act of 1986.

APP/PS1 mice are a transgenic C57BL/6J mouse model co-expressing the Swedish mutation (K595N/M596L) and the deltaE9 PS-1 exon deletion (mutated human presenilin-1). Offspring were tail-snipped and genotyped using PCR. PCR used primers specific for the APP sequence (Forward “GAATTCCGACATGACTCAGG”, Reverse: “GTTCTGCTGCATCTTGGACA”). Mice not expressing the transgene were used as WT controls. Female APP/PS1dE9 mice, aged 6 and 12 months and age-matched WT female C57BL/6J littermate controls (*n* = 5) were used. Mice were fasted for 16 h and blood samples were collected into heparinised tubes, centrifuged for 30 seconds at 13,000× *g* and the resulting plasma were stored at −80 °C prior to metabolomics investigations. Mice were deeply anaesthetised with pentobarbitol and whole mouse brain was also collected and snap-frozen in liquid nitrogen and stored at −80 °C until further use.

### 4.3. Brain Tissue Extraction

The extraction method applied to the samples was identical to our previously published work [22] using Biocrates p180 kits and based upon previous optimisation work undertaken by others [34]. Both human brain and mouse brain samples were collected into individual tubes to avoid cross-contamination, lyophilized, and cryogenically milled to a fine powder. 30 mg (± 0.5 mg) of powdered human PM brain tissue and 15 mg (± 0.5 mg) of powdered mouse brain tissue were weighed and extracted in 100 µL and 50 µL of solvent (85% ethanol and 15% PBS buffer) respectively in a 1.5 mL sterile Eppendorf tube. The samples were shaken (10 min), sonicated (15 min), and centrifuged at (10,000× *g*; 4 °C; 5 min) and the supernatant retained for analysis. 

### 4.4. Bile Acid Quantifications

Quantification of bile acids in both brain tissue extract and plasma were performed using the Biocrates Bile Acids kit (BIOCRATES, Life Science AG, Innsbruck, Austria). The bile acid kit provides simultaneous quantification of 22 bile acids (properties outlined in Table 6). Samples were processed according to the manufacturer’s instructions. Seven calibration standards and a mixture of 10 internal standards are integrated into this kit and three human plasma based quality controls were applied to assess the reproducibility of the assay. Briefly, 10 µL of calibrators, quality controls, plasma samples, and PM brain extracts (prepared as described above) were applied to a 96-well filter plate, which contains isotopic internal standards. All samples were subsequently extracted in methanol (100 µL), diluted with water (60 μL), and analysed using a Waters Acquity UPLC system (Milford, MA, USA) coupled to a triple-quadrupole mass spectrometer (Xevo TQ-S, Waters Corporation, Milford, MA, USA) operating in multiple reaction monitoring (MRM) mode. Metabolite concentrations were calculated with plasma concentrations expressed as nM, and brain expressed as nmol per gram of dry tissue weight. 

## 5. Statistical Analysis

**Figure 1:**
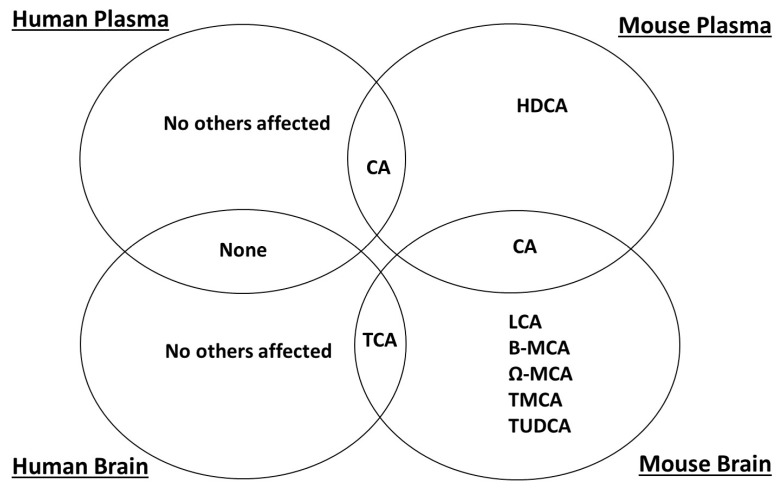
Venn diagram summarising the bile acids significantly altered in each species/sample type.

Results are expressed as mean ± SEM. For statistical analysis, bile acid data were normalised by logarithmic 10 (log 10) transformation, confirmed as normally distributed (Shapiro-Wilk Test, SPPS version 20) and compared by one-way homoscedastic Student’s T-test (Microsoft Excel, 2013). Differences were deemed to be significant if *p* ≤ 0.05). A Venn diagram was compiled (Figure 1) to summarise the differences observed. Receiver operating characteristic (ROC) analysis was performed using online metabolomics tools Metaboanalyst v 3.0 [35]. 

## 6. Conclusions

In conclusion, there appears to be substantial bile acid perturbations both in experimental AD models and in clinical cases of AD. Given the very powerful effects that bile acids have on cellular processes, including their described actions modulating neuronal cell apoptosis, much greater consideration should be afforded to these metabolites when investigating AD.

## Figures and Tables

**Table 1:** Bile acid levels in human plasma.

Bile Acid	Control (*n* = 10)	AD (*n* = 10)	*p*-Value	ROC
**CA**	947 ± 483	156 ± 74	**0.03**	0.77
**CDCA**	2781 ± 2329	312.2 ± 147	0.14	0.62
**DCA**	938 ± 375	638 ± 212	0.28	0.55
**GCA**	281 ± 76	364 ± 67	0.14	0.63
**GCDCA**	1218 ± 261	1118 ± 162	0.47	0.56
**GDCA**	625 ± 125	831 ± 199	0.31	0.58
**GLCA**	42 ± 12	42 ± 10	0.39	0.53
**GLCAS**	484 ± 99	687 ± 126	0.14	0.67
**GUDCA**	141 ± 40	170 ± 84	0.41	0.54
**HDCA**	<LOD	<LOD	-	-
**LCA**	63 ± 19	44 ± 10	0.27	0.63
**α-MCA**	<LOD	<LOD	-	-
**β-MCA**	<LLOQ	<LLOQ	0.08	-
**Ω-MCA**	39 ± 18	15 ± 9	0.25	0.71
**TCA**	39 ± 12	87 ± 25	0.09	0.72
**TCDCA**	147 ± 39	184 ± 56	0.38	0.51
**TDCA**	60 ± 13	106 ± 33	0.21	0.64
**TLCA**	<LLOQ	<LLOQ	-	-
**TLCAS**	<LLOQ	<LLOQ	-	-
**TMCA (α + β)**	<LLOQ	<LLOQ	-	-
**TUDCA**	<LLOQ	<LLOQ	0.40	-
**UDCA**	206 ± 125	101 ± 25	0.31	0.52

Concentrations presented are nM (mean ± SEM). <LOD = Below the limit of detection. <LLOQ = Below the lower limit of quantification. ROC = Receiver operating characteristic. Significant *p*-values are in **bold**.

**Table 2:** Bile acid levels in mouse plasma.

Bile Acid	6 Months				12 Months			
	Control (*n* = 5)	APP/PS1 (*n* = 5)	*p*-Value	ROC	Control (*n* = 5)	APP/PS1 (*n* = 5)	*p*-Value	ROC
**CA**	1811 ± 368	10510 ± 4498	**0.05**	0.80	2934 ± 1247	2099 ± 763	0.45	0.52
**CDCA**	142 ± 44	392 ± 189	0.18	0.68	300 ± 66	197 ± 90	0.17	0.68
**DCA**	970 ± 331	3799 ± 2247	0.08	0.80	1569 ± 431	1642 ± 422	0.37	0.52
**GCA**	<LLOQ	<LLOQ	0.19	-	<LLOQ	<LLOQ	-	-
**GCDCA**	<LOD	<LOD	-	-	<LOD	<LOD	-	-
**GDCA**	<LOD	<LOD	-	-	<LOD	<LOD	-	-
**GLCA**	<LOD	<LOD	-	-	<LOD	<LOD	-	-
**GLCAS**	<LLOQ	<LLOQ	-	-	<LLOQ	<LLOQ	-	-
**GUDCA**	<LLOQ	<LLOQ	-	-	<LLOQ	<LLOQ	-	-
**HDCA**	609 ± 214	405 ± 125	0.20	0.64	1009 ± 226	364 ± 192	**0.04**	0.87
**LCA**	40 ± 10	64 ± 32	0.32	0.56	77 ± 15	88 ± 32	0.48	0.52
**α-MCA**	88 ± 50	211 ± 103	0.13	0.65	391 ± 176	136 ± 73	0.37	0.72
**β-MCA**	3132 ± 977	8780 ± 3781	0.10	0.76	4271 ± 1706	4716 ± 1258	0.30	0.60
**Ω-MCA**	3068 ± 1035	5246 ± 2189	0.24	0.68	2368 ± 1213	1235 ± 332	0.42	0.52
**TCA**	865 ± 251	9473 ± 9007	0.36	0.60	1258 ± 244	1051 ± 331	0.31	0.53
**TCDCA**	52 ± 10	610 ± 575	0.36	0.58	87 ± 15	68 ± 17	0.18	0.70
**TDCA**	143 ± 40	912 ± 822	0.34	0.52	159 ± 11	188 ± 43	0.40	0.60
**TLCA**	<LLOQ	<LLOQ	-	-	<LLOQ	<LLOQ	-	-
**TLCAS**	<LLOQ	<LLOQ	-	-	<LLOQ	<LLOQ	-	-
**TMCA (α + β)**	1529 ± 322	5406 ± 4260	0.38	0.52	1326 ± 318	1408 ± 384	0.44	0.52
**TUDCA**	347 ± 72	1723 ± 1582	0.42	0.68	990 ± 437	513 ± 221	0.22	0.76
**UDCA**	363 ± 150	1348 ± 784	0.10	0.72	693 ± 179	838 ± 287	0.26	0.52

Concentrations presented are nM (mean ± SEM). <LOD = Below the limit of detection. <LLOQ = Below the lower limit of quantification. ROC = Receiver operating characteristic. Significant *p*-values are in **bold**.

**Table 3:** Bile acid levels in human brain tissue.

Bile Acid	Control (*n* = 10)	AD (*n* = 10)	*p*-Value
**CA**	0.16 ± 0.04	0.24 ± 0.1	0.39
**CDCA**	0.33 ± 0.11	0.65 ± 0.32	0.41
**DCA**	0.37 ± 0.11	1.84 ± 1.39	0.47
**GCA**	0.13 ± 0.03	0.10 ± 0.02	0.25
**GCDCA**	0.19 ± 0.08	0.14 ± 0.05	0.49
**GDCA**	0.06 ± 0.02	0.10 ± 0.04	0.46
**GLCA**	<LOD	<LOD	-
**GLCAS**	<LLOQ	<LLOQ	-
**GUDCA**	<LLOQ	<LLOQ	-
**HDCA**	<LLOQ	<LLOQ	-
**LCA**	0.06 ± 0.02	0.05 ± 0.01	0.44
**α-MCA**	<LOD	<LOD	-
**β-MCA**	<LOD	<LOD	-
**Ω-MCA**	<LLOQ	<LLOQ	-
**TCA**	0.06 ± 0.02	0.01 ± 0.006	**0.01**
**TCDCA**	0.11 ± 0.03	0.04 ± 0.01	0.07
**TDCA**	<LLOQ	<LLOQ	-
**TLCA**	<LOD	<LOD	-
**TLCAS**	<LOD	<LOD	-
**TMCA (α + β)**	<LOD	<LOD	-
**TUDCA**	<LOD	<LOD	-
**UDCA**	0.06 ± 0.01	0.16 ± 0.09	0.31

Concentrations presented are nmol/g (mean ± SEM). <LOD = Below the limit of detection. <LLOQ = Below the lower limit of quantification. Significant *p*-values are in **bold**.

**Table 4:** Bile acid levels in mouse brain.

Bile Acid	6 Months			12 Months		
	Control (*n* = 5)	APP/PS1 (*n* = 5)	*p*-value	Control (*n* = 5)	APP/PS1 (*n* = 5)	*p*-Value
**CA**	0.09 ± 0.01	0.10 ± 0.02	0.47	0.28 ± 0.08	0.08 ± 0.02	**0.02**
**CDCA**	<LOD	<LOD	-	<LOD	<LOD	-
**DCA**	0.07 ± 0.01	0.09 ± 0.02	0.35	0.05 ± 0.003	0.04 ± 0.01	0.17
**GCA**	<LOD	<LOD	-	<LOD	<LOD	-
**GCDCA**	<LLOQ	<LLOQ	-	<LLOQ	<LLOQ	-
**GDCA**	<LOD	<LOD	-	<LOD	<LOD	-
**GLCA**	<LOD	<LOD	-	<LOD	<LOD	-
**GLCAS**	<LOD	<LOD	-	<LOD	<LOD	-
**GUDCA**	<LOD	<LOD	-	<LOD	<LOD	-
**HDCA**	<LOD	<LOD	-	<LOD	<LOD	-
**LCA**	0.01 ± 0.01	0.04 ± 0.01	**0.05**	0.04 ± 0.01	0.05 ± 0.02	0.47
**α-MCA**	<LOD	<LOD	-	<LOD	<LOD	-
**β-MCA**	0.08 ± 0.02	0.07 ± 0.02	0.37	0.24 ± 0.06	0.08 ± 0.04	**0.02**
**Ω-MCA**	0.05 ± 0.01	0.03 ± 0.01	0.22	0.08 ± 0.02	0.03 ± 0.01	**0.05**
**TCA**	0.10 ± 0.03	0.06 ± 0.03	0.11	0.16 ± 0.07	0.04 ± 0.02	**0.04**
**TCDCA**	<LOD	<LOD	-	<LOD	<LOD	-
**TDCA**	<LOD	<LOD	-	<LOD	<LOD	-
**TLCA**	<LOD	<LOD	-	<LOD	<LOD	-
**TLCAS**	<LOD	<LOD	-	<LOD	<LOD	-
**TMCA (α + β)**	0.14 ± 0.02	0.08 ± 0.03	**0.05**	0.25 ± 0.07	0.04 ± 0.01	**0.002**
**TUDCA**	0.02 ± 0.01	0.02 ± 0.002	0.33	0.03 ± 0.01	0.01 ± 0.001	**0.02**
**UDCA**	<LOD	<LOD	-	<LOD	<LOD	-

Concentrations presented are nmol/g (mean ± SEM). <LOD = Below the limit of detection. <LLOQ = Below the lower limit of quantification. Significant *p*-values are in **bold**.

**Table 5:** Summary of characteristics for human brain and plasma samples.

Sample type	Demographics	AD (*n* = 10)	Control (*n* = 10)
**Human Brain**	Age (years: mean (sd))	76.2 (2.3)	74.5 (3.5)
	Range (min-max)	71–79	68–80
	Gender F:M	5:5	4:6
**Human Plasma**	Age (years: mean (sd))	76.3 (5.6)	77.6 (7.5)
	Range (min-max)	70–88	66–87
	MMSE (mean (sd))	21.9 (4.8)	29.3 (0.8)
	Gender F:M	6:4	5:5

MMSE – Mini-Mental State Examination.

**Table 6:** Bile acids measured in this study.

	Bile Acid	Abbreviation	Empirical Formula	Molecular Mass	IUPAC Name
1	Cholic acid	CA	C24H40O5	408.57	(3α,5β,7α,12α)-3,7,12-Trihydroxycholan-24-oic acid
2	Chenodeoxycholic acid	CDCA	C24H40O4	392.57	(3α,5β,7α,8ξ)-3,7-Dihydroxycholan-24-oic acid
3	Deoxycholic acid	DCA	C24H40O4	392.57	(3α,5β,12α)-3,12-Dihydroxycholan-24-oic acid
4	Glycocholic acid	GCA	C26H43NO6	465.62	(3α,5β,7α,8ξ,12α,20R,24Z)-3,7,12,24-Tetrahydroxycholan-24-ylidene]glycine
5	Glycochenodeoxycholic acid	GCDCA	C26H43NO5	449.62	(3α,5β,7α,8ξ,20R,24Z)-3,7,24-Trihydroxycholan-24-ylidene]glycine
6	Glycodeoxycholic acid	GDCA	C26H43NO5	449.62	(3α,5β,12α,20R,24Z)-3,12,24-Trihydroxycholan-24-ylidene]glycine
7	Glycolithocholic acid	GLCA	C26H43NO4	433.62	3α-hydroxy-5β–cholan-24-oylglycine
8	Glycolithocholic acid sulphate	GLCAS	C26H42NO7S	512.27	3α-hydroxy-5β-cholan-24-oyl)-glycine 3-sulphate
9	Glycoursodeoxycholic acid	GUDCA	C26H43NO5	449.31	3α,7β-dihydroxy-5β–cholan-24-oylglycine
10	Hyodeoxycholic acid	HDCA	C24H40O4	392.57	(3α,5β,6α)-3,6-Dihydroxycholan-24-oic acid
11	Lithocholic acid	LCA	C24H40O3	376.57	(3α,5β)-3-Hydroxycholan-24-oic acid
12	α-muricholic acid	α-MCA	C24H40O5	408.57	(3α,5β,6β,7α)-3,6,7-Trihydroxycholan-24-oic acid
13	β-muricholic acid	β-MCA	C24H40O5	408.57	(3α,5β,6β,7β)-3,6,7-Trihydroxycholan-24-oic acid
14	Ω-muricholic acid	Ω-MCA	C24H40O5	408.57	(3α,5β,6α,7β)-3,6,7-Trihydroxycholan-24-oic acid
15	Taurocholic acid	TCA	C26H45NO7S	515.70	(3α,5β,7α,8ξ,12α,20R,24Z)-3,7,12-Trihydroxy-N-(2-sulphoethyl)cholan-24-imidic acid
16	Taurochenodesoxycholic acid	TCDCA	C26H45NO6S	499.70	2-{[(3α,5β,7α,8ξ,9ξ,14ξ)-3,7-Dihydroxy-24-oxocholan-24-yl]amino}ethanesulphonic acid
17	Taurodeoxycholic acid	TDCA	C26H45NO6S	499.70	α,12α-dihydroxy-5β–cholan-24-oyltaurine
18	Taurolithocholic acid	TLCA	C26H45NO5S	483.70	(3α,5β,20R,24Z)-3-Hydroxy-N-(2-sulphoethyl)cholan-24-imidic acid
19	Taurolithocholic acid sulphate	TLCAS	C26H45NO8S2	563.76	(3α,5β,20R,24Z)-N-(2-Sulphoethyl)-3-(sulphooxy)cholan-24-imidic acid
20	Tauromuricholic acid (α and β)	TMCA (α + β)	C26H45NO7S	515.70	3α,6β,7α/β -trihydroxy-5β–cholan-24-oyltaurine
21	Tauroursodeoxycholic acid	TUDCA	C26H45NO6S	499.70	2-{[(3α,5β,7β)-3,7-Dihydroxy-24-oxocholan-24-yl]amino}ethanesulphonic acid
22	Ursodeoxycholic acid	UDCA	C24H40O4	392.57	(3α,5β,7β)-3,7-Dihydroxycholan-24-oic acid

## Supplementary Materials

The following are available online at http://www.mdpi.com/2218-1989/7/2/28/s1, Table S1: Participant demographics and clinical characteristics for human plasma samples. Table S2: The sample cohort provided by Newcastle Brain Trust and the information pertaining to the post-mortem tissue.
