# Supplementary material for: Metabolomic Profiling of Bile Acids in Clinical and Experimental Samples of Alzheimer’s Disease
Source: Metabolites. 2017 Jun 17;7(2):28. doi: 10.3390/metabo7020028 (PMC5487999; doi:10.3390/metabo7020028)
Supplement: Supplementary file 1 [file metabolites-07-00028-s001.pdf]

Supplementary Material

Supplementary Table 1. Participant demographics and clinical characteristics for human plasma samples

AD					Control				
Gender	Age	Education (years)	Initial MMSE	APOE4	Gender	Age	Education (years)	Initial MMSE	APOE4
F	73	12	13	44	F	84	12	30	na
F	70	10	24	na	F	87	20	30	na
F	88	10	24	34	F	76	12	28	33
F	75	10	24	33	M	75	14	30	34
F	77	10	25	33	M	73	10	29	33
M	70	12	13	34	M	86	10	30	34
F	77	9	24	34	M	72	10	29	33
M	83	14	23	44	F	71	19	30	33
M	75	17	26	34	M	86	10	28	23
M	75	10	23	33	F	66	13	29	34

Supplementary Table 2. The sample cohort provided by Newcastle Brain Trust and the information pertaining to the post-mortem tissue.

AD				Control			
Gender	Age	PM Delay	Braak Stage	Gender	Age	PM Delay	Braak Stage
F	71	67	5	F	73	59	1
M	75	54	5	F	72	24	0
F	79	27	6	M	78	48	1
F	75	21	6	M	77	42	1
M	78	49	6	M	73	25	0
F	77	63	6	F	68	38.75	0
M	78	37	6	F	74	39.5	1
M	75	39.5	5	M	80	45.75	0
F	77	19	6	M	73	23	0
M	77	19	6	M	77	10-11	0
